# Seed Rain and Seed Bank Reveal that Seed Limitation Strongly Influences Plant Community Assembly in Grasslands

**DOI:** 10.1371/journal.pone.0103352

**Published:** 2014-07-24

**Authors:** Bryndís Marteinsdóttir

**Affiliations:** The Department of Ecology, Environment and Plant Sciences, Stockholm University, Stockholm, Sweden; University of Saskatchewan, Canada

## Abstract

Dispersal is an important factor in plant community assembly, but assembly studies seldom include information on actual dispersal into communities, i.e. the local propagule pool. The aim of this study was to determine which factors influence plant community assembly by focusing on two phases of the assembly process: the dispersal phase and the establishment phase. At 12 study sites in grazed ex-arable fields in Sweden the local plant community was determined and in a 100-m radius around the centre of each site, the regional species pool was measured. The local seed bank and the seed rain was explored to estimate the local propagule pool. Trait-based models were then applied to investigate if species traits (height, seed mass, clonal abilities, specific leaf area and dispersal method) and regional abundance influenced which species from the regional species pool, dispersed to the local community (dispersal phase) and which established (establishment phase). Filtering of species during the dispersal phase indicates the effect of seed limitation while filtering during the establishment phase indicates microsite limitation. On average 36% of the regional species pool dispersed to the local sites and of those 78% did establish. Species with enhanced dispersal abilities, e.g. higher regional abundance, smaller seeds and dispersed by cattle, were more likely to disperse to the sites than other species. At half the sites, dispersal was influenced by species height. Species establishment was however mainly unlinked to the traits included in this study. This study underlines the importance of seed limitation in local plant community assembly. It also suggests that without information on species dispersal into a site, it is difficult to distinguish between the influence of dispersal and establishment abilities, and thus seed and microsite limitation, as both can be linked to the same trait.

## Introduction

Dispersal into plant communities has been identified as one of the major factors influencing plant community assembly (i.e. seed limitation [1], [2]). The assembly process is then further affected by the species' abilities to establish under the abiotic and biotic conditions at a site (i.e. microsite limitation; [3], [4]). The influence of species dispersal and establishment abilities on plant community assembly can be studied using null models. Null models investigate if a local plant community is a random or non-random sample from a regional species pool. These models often explore how species are assembled into communities based on functional traits i.e. measurable morphological, physiological and life history characteristics of the plant that influence plant fitness through their effect on reproduction, growth and survival [5], [6]. Differentiation between seed and microsite limitation in these models is then based on a particular trait showing non-random assembly [2]. For example, a non-random distribution of a dispersal trait would indicate seed limitation. If a trait related to species establishment or competition abilities exhibit a non-random distribution, this would indicate microsite limitation. However, some traits, such as seed size, can be both linked to species dispersal [7] and establishment [8]–[10], complicating the distinction between seed and microsite limitation. This complication could be resolved by including measurements on species dispersal into communities in these models.

The current study was conducted in grazed ex-arable fields. These grassland systems are former crop fields that have been transformed to pastures relatively recently, i.e. within the last ca. 20–40 years [11]. A previous study at the study sites used trait-based models to investigate how plant species were filtered from the regional species pool into local communities [12] and differentiated between seed and microsite limitation based on which traits exhibited non-random assembly. It was concluded that the filtering was partly linked to dispersal methods, species abundance in the regional species pool, and local microsite limitation. In the current study the species dispersal into the study sites was estimated (i.e. the local propagule pool) and the assembly process then divided up to two phases: the dispersal phase and the establishment phase. The main aim was to determine which factor influenced plant community assembly in each phase. Specially, two questions were addressed 1) What is the relative importance of seed and microsite limitation in plant community assembly? 2) How are species filtered from one species pool to another? Exploration of the linkage between the species that are found in a region surrounding a site (the regional species pool), the species that disperse to a site (the local propagule pool) and the species that establish (the local species pool) [12] allows for more accurate testing on factors that influence local species assembly, as no prior assumptions have to be made on which traits are linked to species dispersal and which to species establishment.

To assess the relative importance of seed and microsite limitation in plant community assembly, I compared the proportion of species filtered out in each phase. The proportion of species in the regional species pool that disperse to a site will indicate the strength of the seed limitation while the proportion of species in the local propagule pool that establish will imply the strength of the microsite limitation. Also, investigation on where the species in the local propagule pool come from will give indication of the role of dispersal (and thus seed limitation) in maintaining the diversity at the sites (cf. [13]).

To determine how species were filtered from one species pool to another, trait-based models were applied for each phase. If the assembly is deterministic during the dispersal phase then species with traits that enhance species dispersal e.g. smaller seed size [7] are expected to overcome the seed limitation. However, if species filtering in the dispersal phase is a neutral process, then species that are common in at the regional scale should also be common at the local scale, as more abundant species will have more flowering individuals and larger seed production and thus disperse better than rare species [14]. Non-random filtering of species between the local propagule pool and the local community indicates abiotic (i.e. environmental filtering) or biotic (i.e. species interaction filtering) microsite limitation. Trait convergence (underdispersion) will imply environmental filtering [15], [16], as only species with particular traits can establish under the local environmental conditions. Trait divergence (overdispersion) will reflect the effect of species competition as species with similar traits are expected to have similar resource requirements and thus compete more intensely (i.e. limiting similarity [5], [17]). The convergence of traits correlated with competitive ability can also indicate species interaction filtering (e.g. [18], [19], [20]), as less competitive species are filtered out. Furthermore, some authors have suggested that by being similar enough species escape the rule of limiting similarity and coexist [21], [22], causing convergence in traits related to species establishment and persistence. In addition, as the ex-arable fields in this study are relatively young ecosystems, the local species assembly should be representative of what is found in the surrounding region today. A positive correlation between species richness in the regional species pool and the local propagule pool should thus indicate the effect of neutral processes, as the local species richness is simply a product of the richness in the regional species pool (i.e. species pool hypothesis [23]).

## Methods

### Site selection

The study was conducted in a cultivated landscape, with a mosaic of arable fields, ex-arable fields, semi-natural grasslands and forests, located in Nynäs nature reserve, southeastern Sweden (58°49′N, 17°24′E) [11]. Within the area, 12 sites in species poor ex-arable fields were chosen with a distance between the sites of at least 300 m. All the sites were grazed with livestock (sheep or cattle). At each site a plot of 10×10 m was laid out in a homogeneous area more than 6 m from the field edge. Permission for conducting field studies in the study sites were obtained from Öknaskolan, the institution responsible for the management of the nature reserve. This field study did not involve any protected or endangered species.

### Seed rain and seed bank sampling

To estimate the viable seed bank, soil samples were collected in early April 2011, before the first spring plants started to flower. From each of the 12 study sites, 20 samples were randomly collected from the 10×10 m plot. Each sample consisted of four soil cores mixed together, taken with an auger, 2 cm in diameter, down to 5 cm depth, so at each site 80 soil cores were collected, creating 20 seed bank samples. In total of 240 seed bank samples were taken. The samples were stored in darkness at ca. 4°C until they were processed.

For estimation on the local seed rain, 20 seed traps were laid out in the 10×10 m plots at each site, in late May 2011. The seed traps were 8×8×10 cm pots filled with potting soil. The seed traps were buried into the ground so that their edge was on ground level. In October 2011 the first pots were replaced with new plots. The second pots were collected in late May 2012.

### Germination of samples

Processing of the seed bank samples were done in October 2011 and of the seed rain samples immediately after collection of seed traps in October 2011 and May 2012. Each seed bank sample was first washed through a 40 mm mesh to remove large stones and vegetative parts and a 2 mm mesh to remove small soil particles. These procedures are in accordance with Heerdt *et al.* [24]. The seed bank and seed rain samples were spread onto a 12×10.5×7 cm tray filled with layer of potting soil covered by a 0.5 cm layer of sterile sand. Before spreading the seed rain samples all stones and leaves were removed and already established seedlings transplanted or identified. The samples were kept in a greenhouse at the Department of Ecology, Environment and Plant Sciences at Stockholm University, with the day temperature at ca. 17°C and night temperature ca. 14°C, and with 15 hours of light per day. The samples were watered as needed. The germination of seedlings was monitored every other week and each seedling was either identified and removed or transplanted for later identification. After each removal the soil was moved around to stimulate germination. The seed bank samples were monitored until May 2012 and the seed rain samples until August 2012. Seed rain samples collected in October 2011 were placed outside in the middle of December for two months, so that the remaining seeds would experience winter. In mid-February 2012 the pots were placed back into the greenhouse and germination followed. As seed rain samples collected in May had already experienced winter, there was no need of freezing to stimulate germination.

Not all species could be identified to the species level and in those cases species were grouped together i.e. *Poa compressa/pratensis* and *Alchemilla* spp. (for detailed species list with groups see Table S1). Hereafter these groups will be considered as species.

In the greenhouse 24 control plots were laid out randomly, to account for contamination from the potting soil and incoming seed rain. Few seedlings germinated in the control plots, mostly *Geranium robertianum,* which is a common weed in the greenhouse and *Betula* spp. which has highly dispersed seeds. Both of those species were omitted from the analysis of the seed bank and seed rain data.

### Vegetation sampling

Within each site, species occurrence was estimated in the 10×10 m plots. Abundance was estimated (on a scale from 0–8) in four 1×2 m subplots within each 10×10 m plot. Species found in the 10×10 m plot but not in the subplots were given an arbitrary abundance value of 0.1. The local community was defined as all vascular plant species growing within the 10×10 m plot. Around each site, the regional species pool (richness and abundance) was determined in a 100 m radius from each site, including the species found within each 10×10 m plot. The area within the radius was divided up in to vegetation types, and the proportion of each vegetation type estimated with aerial photographs in ArcGIS. Within each vegetation type the species presence was recorded and the commonness of each species (very common, common, rare) estimated. For more information on measurements on species abundance in the four sub-plots and on the regional species pool see Marteinsdóttir and Eriksson [12]. For direct comparison with the seed rain and seed bank data, plant species in the local community and regional species pool were grouped together, as explained before.

Nomenclature follows Mossberg and Stenberg [25].

### Statistical analysis

In total 240 seed bank samples and 198 summer and 185 winter seed rain samples were analysed. All 20 seed bank samples within a site were compiled before analyses, to represent an estimation for the site seed bank (N = 12). Due to disturbance the sample size of the winter and summer seed rain within sites was not always equal. Seed rain samples were therefore standardized to allow for estimations of the total yearly seed rain within a site. For each species within a site the cumulative number of seedlings was multiplied with the proportion of samples recovered from the site (out of 20). This was done separately for the winter and summer seed rain. The standardized site summer and winter seed rain was then summarized to yield a total site seed rain for each species (N = 12).

The regional species pool of a site was defined as all species found in the region excluding trees and non-seed bearing plants. Trees were excluded as they rarely establish in ex-arable fields, and if they did, they were usually removed by the land managers. The local propagule pool of a site was defined as all species found in the local seed rain and seed bank as well as all species found in the local community at a site. The measured seed rain at a site only represents the one year species dispersal, and is therefore likely to overestimate the effect of seed limitation. The seed bank represents dispersal through time and similarly the already established vegetation represents which species have been able to disperse to and establish in the community. By including the seed bank and established vegetation in the local propagule pool, overestimation of the seed limitation is minimized. Trees and non-seed bearing plants were also excluded from the local propagule pool and local community.

To explore the relative importance of seed and microsite limitation, the fraction of species filtered out between pools was calculated: regional species pool – local propagule pool – local community. To test if there was a significant relationships between species richness in the regional species pool and the local propagule pool/local community/seed rain/seed bank a Monte Carlo simulations were used [26], [27]. For each simulations a random number, from 0 to the number of species in the regional species pool was obtained, to represent the sites small-scale species-richness (local propagule pool, local community, seed rain and seed bank). This was done for all the 12 sites and a Spearman's correlation coefficient then calculated for the relationship between this random site local richness and the observed regional richness. This was repeated 4999 times and p-values (one-tailed test) were calculated as the proportion of simulated correlation coefficients that were equal or higher than the observed correlation coefficient (obs. rho).

Monte Carlo simulations were also used to determine if species traits influenced the filtering of species from the regional species pool into the local propagule pool/seed rain/seed bank and from the local propagule pool into the local community. The test statistics used to analyse the filtering of traits between different species pools were mean trait values, trait values variance and standard deviation of the nearest trait distance. Mean trait values test if species that were filtered into a small scale species pool had on average higher or lower trait values than expected by chance. Trait values variance and standard deviation of the nearest trait distance both indicate how similar or dissimilar traits are within a site. If the variance or trait distance is higher than expected by chance it indicates trait convergence (underdispersion) but trait divergence (overdispersion) if the variance or trait distance is lower than expected by chance. For each site, the test statistic was compared to distributions of simulated values calculated from 4999 virtual values, generated by reassigning species and associated traits, randomly and without replacement from the regional species pool into the local propagule pool/seed rain/seed bank and from the local propagule pool into the local community. The number of species in each random assembly was equal to the number in the observed local propagule pool/local community/seed bank/seed rain. The proportion of Monte Carlo-derived values that were either lower or higher than the observed trait values were used to calculate the p-values and the difference was determined significant if either of the p-values were under 0.025 (two-tailed). Mean trait values and site trait variance were both calculated where species were chosen at random from the regional species pool into the local propagule pool/seed rain/seed bank and where the probability of species to be chosen was linked to the species abundance in the regional species pool. Standard deviation of the nearest trait distance were only calculated where species were chosen at random from the regional species pool. Simulations for the filtering between the local propagule pool and the local community were all based on random probabilities. Five functional traits were used in these simulations: seed mass, specific leaf area (SLA), height, dispersal method and clonal growth index (CGI). CGI and height are often associated with species persistence, SLA with species establishment and dispersal method with species dispersal. Seed mass has both been linked to species dispersal and establishment [28]. As dispersal method was, unlike the other traits, based on categorized values, only the site mean trait value were calculated, using the proportion of each category within a site/simulation. In addition to the five traits, species abundance in the regional species pool was used in models exploring the filtering between the regional species pool and the local propagule pool. For abundance only site mean trait values were calculated. For more information on this method and the traits explored see Marteinsdóttir and Eriksson [12].

A Spearman's rank correlation coefficient was used to explore the correlation between the abundance of species in the local seed rain/seed bank and local community at each site and between the regional species pool and the local seed rain/seed bank/community at each site.

Statistical analyses were conducted using R 3.0.2 for Windows (available from http://cran.r-project.org). For raw data used in these analyses see Table S2.

## Results

A total of 1489 seedlings of 57 species were found in the seed bank samples (N = 240). In the seed rain 5915 seedlings of 86 species were found, there were 5579 seedlings of 73 species in the summer seed rain (N = 215) and 336 seedlings of 38 species in the winter seed rain (N = 199). The average seed density in the seed rain was 42 seeds/dm^2^. In the local community (10×10 m plots) 99 species were identified, 278 species in the regional species pools and 138 species in the local propagule pools (i.e. the seed rain, seed bank and local community). In the whole study, in total of 287 species were found.

**Figure 1:**
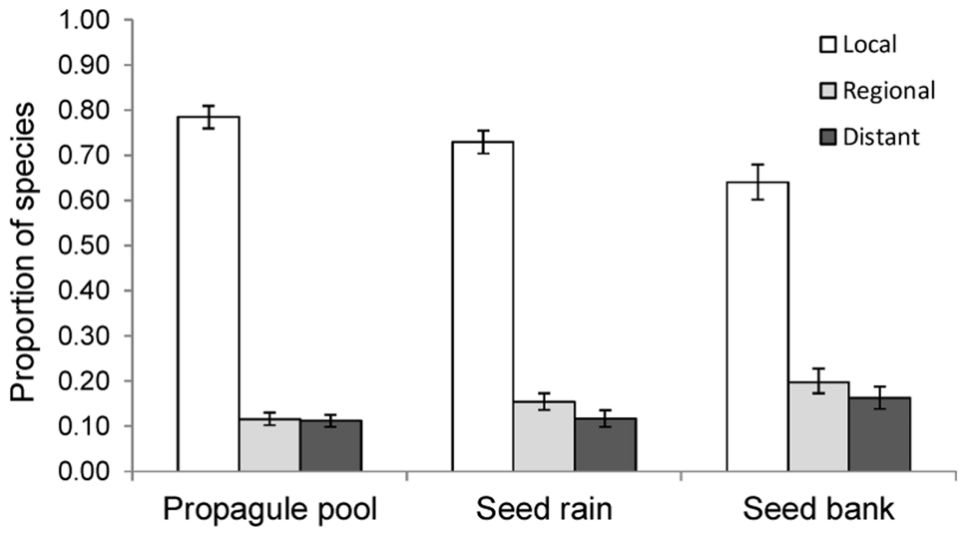
The origin of species that disperse to a site. The proportion of species (±SE) in the local propagule pool, the seed rain and the seed bank, found in the local community (Local), only found in the regional species pool but not the local community (Regional), and not found in the regional species pool, thus indicating long distance dispersal (Distant). The proportions are average proportions from the 12 ex-arable fields in Nynäs, Sweden.

For species in the regional species pool surrounding each site on average 36% (SE±1.7%) were found in the local propagule pool and on average 78% (SE±2.5%) of species in the local propagule pool were found in the local community. A majority of species in the local propagule pool were already a part of the local community at a site, whereas 12% came from the regional species pool surrounding each site and 11% came from further away (Fig. 1).

There was a positive relationship between species richness in the regional species pool and the species richness in the local community (obs. rho = 0.69, p = 0.016), the local propagule pool (obs. rho = 0.65, p = 0.023) and the seed bank (obs. rho = 0.68, p = 0.016), but there was no relationship between species richness in the regional species pool and species richness in the seed rain (obs. rho = 0.40, p = 0.14).

At some sites there was a positive correlation between abundance in the regional species pool and the local community (9 sites), the seed bank (5 sites), and the seed rain (6 sites). Abundance in the local community was positively correlated with abundance in the seed bank at three sites and in the seed rain at all sites (Table S3).

**Figure 2:**
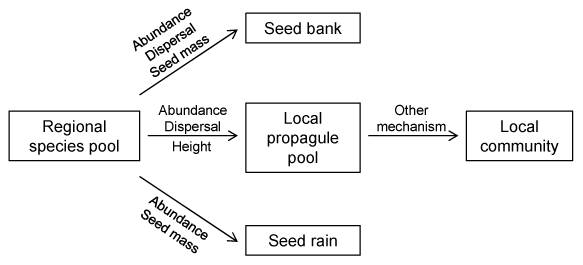
The filtering of species between different species pools. The influence of species trait and abundance on the filtering of species from the regional species pool surrounding a site into the local propagule pool, the local seed rain and the local seed bank and from the local propagule pool into the local community. Names above the arrows indicate which factor influenced the filtering in majority of the 12 ex-arable fields studied, in Nynäs nature reserve, Sweden. Abundance: indicates that species abundance in the regional species pool influenced dispersal into a site. Dispersal method: indicates that species with unassisted dispersal were more likely to disperse to a site. Seed mass: indicates that species with lower seed mass were more likely to disperse to a site. Other mechanism: indicates that species filtering was not highly influenced by any of the traits explored in this study, i.e. clonal growth index, dispersal method, height, seed mass and specific leaf area.

Species in the local propagule pool were those from the regional species pool that were abundant and had unassisted dispersal. Also, at half the sites there was trait convergence in height between the regional species pool and local propagule pool. Species in the seed rain and seed bank were those from the regional species pool that were abundant and had small seeds. In addition, at some sites species with unassisted dispersal were proportionally more often found in the local seed bank (8 sites) and seed rain (4 sites). There were no clear filtering effects between the local propagule pool and the local community (Fig. 2, Table 1).

**Table 1:** A summary of results from randomization tests.

	Abundance	Height	Seed mass	CGI	SLA	Dispersal
**Regional pool to propagule pool**						
Mean values (EP)	12	±3	−1	3	−1	11
Mean values (WP)	NA	±2	−3	2	0	6
Variance (EP)	NA	−6	0	0	−3	NA
Variance (WP)	NA	−5	−1	0	−3	NA
SdNTD	NA	−5	0	0	−2	NA
**Propagule pool to local community**						
Mean values	NA	2	3	1	0	2
Variance	NA	−2	0	0	−3	NA
SdNTD	NA	−1	0	0	0	NA
**Regional pool to seed bank**						
Mean values (EP)	11	0	−10	0	0	8
Mean values (WP)	NA	0	−8	0	0	2
Variance (EP)	NA	−1	−6	−3	0	NA
Variance (WP)	NA	−1	−4	−3	0	NA
SdNTD	NA	−1	−2	0	1	NA
**Regional pool to seed rain**						
Mean values (EP)	12	0	−7	0	0	4
Mean values (WP)	NA	0	−7	0	0	1
Variance (EP)	NA	−3	−6	−1	0	NA
Variance (WP)	NA	−2	−4	−1	0	NA
SdNTD	NA	−1	−5	0	0	NA

The table displays the number (out of 12) of ex-arable field sites that had non-random assembly of a given trait. Species assembly between the regional species pool (regional pool) and the local propagule pool (propagule pool), local seed rain and local seed bank were explored as well as the assembly between the local propagule pool and the local community. The traits used were species abundance in the regional species pool (Abundance), mean height, seed mass, specific leaf area (SLA), clonal growth index (CGI) and dispersal method. The statistics were mean trait values (mean values), standard deviation of the nearest trait distance (sdNTD), site trait variance (variance). Mean trait values and site trait variance for assembly from the regional species pool were both calculated where species were chosen at random from the regional species pool into the local propagule pool/seed rain and seed bank (EP) and where the probability of species to be chosen was linked to the species abundance in the regional species pool (WP). For dispersal method the number represent sites with higher proportion of unassisted dispersal than expected from a random assemble. – in front of a number indicate trait convergence, + indicates trait divergence. NA represent associations not explored.

## Discussion

This study shows that seed limitation is a strong determinant of plant community assembly, as only a small proportion of all possible colonizers found in the area surrounding a site did actually disperse there. Microsite limitation also influences the assembly, but to a lesser degree, as not all species that reach a site did establish.

Most species only disperse a short distance [29] and here a majority of species in the seed rain and seed bank were also found in the local community. There was however a small but constant input of non-local species, both from the regional species pool and from other long distance source pools. Even though rare, this long distance dispersal may be very important for community assembly [13], [30]. New species are dispersing to the sites and under the right establishment conditions these long distance dispersers might colonize, changing the community dynamics. With time more and more non-local species will reach the site and establish reducing the effect of dispersal limitation. In addition, the species richness in the local propagule pool was influenced by the species richness in the region. Thus, processes that shape and determine the regional species richness, will through dispersal, influence local richness in these ex-arable fields. This is in accordance with the species pool hypothesis [23]. However, when the components of the local propagule pool are separated, the relationship between regional and local richness is only found in relation to the seed bank and established vegetation but not for the seed rain. While the measured seed rain only represents one year of seed dispersal, and is dependent on current conditions, i.e. which species produce seed this year, the seed bank and established vegetation can be seen as the accumulation of seed rain over several year and should therefore represent the source community better [31].

Which species from the region dispersed to a site was partly determined by the species abundance in the regional species pool. Also, species that were abundant in the regional species pool were also abundant in the local community. This is consistent with neutral community models that predict that common species, that will produce more propagules, are more likely to disperse to and be common at other sites, than rare species [14]. A similar association between species abundance in the region and local occurrence has been found in grazed grasslands in England [32]. Furthermore, the positive relationship between abundance in the local community and the local seed rain is also consistent with neutral community models. Species without any special dispersal mechanism were more likely to reach a site than other species. These species are often grasses and other species that are commonly ingested by grazers [33], and grazing animals have been found to be important dispersal method in grazed communities [34], [35]. Previous study in the study system examining the filtering between the regional species pool and local community also found that species with high regional abundance and species that dispersed by grassers were more likely to colonize the sites than other species [12]. In this study there were indications that species with lower seed mass and thus higher seed production [7], [36], [37] and longer dispersal distance [29], [38] were more common in the seed rain and seed bank than expected by random. Study on community assembly in grasslands in central Europe have found similar patterns [39], while the previous study in these fields [12] and other studies in ex-arable fields in Sweden [3] and Canada [40] have concluded that colonization abilities of plants are unlinked to seed mass. Here, seed mass was not only reflecting the abilities of species to disperse to a site. At three sites species with higher seed mass were more likely to establish from the local propagule pool than other species, reflecting higher establishment success of larger seeds [7], [37]. The lack of the filtering effect of seed mass when studying the assembly from the regional species pool into local community found in previous studies might therefore result from these two contrasting processes cancelling each other out, rather than stochastic assembly.

During the dispersal phase, at half the sites there was a trait convergence in height, a trait that is usually associated with species establishment. A study on filtering between the regional species pool and a local community at the study sites also found trait convergence at the same sites, but as height is usually associated with species persistence, it was concluded that this indicated local microsite limitation [12]. Dividing the assembly process into two phases, reveals that this filtering is mainly occurring during species dispersal into a community, but not during establishment. Traits, like height, that improve species establishment on the local scale might also enhance species performance at the regional scale, increasing regional reproductive success and dispersal. These results indicate that height might be representing characteristics of the plants that both influence species establishment and dispersal. This was however only detected when the colonization process was divided into two steps.

In this study, species were mostly randomly assembled from the local propagule pool to the local community. However, a seed sowing and transplantation experiment at the sites, did find that local species have some advantage over non local species, indicating a deterministic filtering during the establishment phase (Marteinsdóttir, unpublished data). Results from studies on community assembly using traits and null models do seldom detect a non-random co-occurrence of plant species, but whether this is a true pattern or a methodological shortcoming has been debated [2]. The lack of deterministic assembly between the local propagule pool and the local community seen here might be because the filtering is affecting traits not including in this study. It might also be because microsite limitation is a neutral process, working equally on all species

## Conclusions

This study underlines the influence of regional processes on local plant community assembly (cf. [41]) which was strongly determined by species regional abundance and traits that enhanced seed production and dispersal abilities of species in the region. There was however limited evidence for local processes affecting the assembly. This importance of regional processes is however only fully revealed when the local propagule pool is included in the study. Without information on which species disperse to a site, it is hard to determine if deviation from random assembly is caused by factors that influence which species disperse to the site or on microsite limitation. Also, contrasting deterministic processes working on traits during the dispersal and establishment phase, can cancel each other out, giving the impression of random assembly. While measuring the local propagule pool at a site is a time-consuming process, the results from this study indicate that knowledge about which species are actually dispersing to a site is necessary to further our understanding on plant community assembly.

## Supporting Information

Table S1 **The occurrence of species in different species pools.** The occurrence of species in the regional species pool, the local propagule pool, the local community (vegetation), the seed rain and the seed bank. The number represent at how many of the 12 ex-arable fields in Nynäs, Sweden the species was found.
(PDF)

Table S2 **Site species abundance in the regional species pool, seed rain, seed bank and local community.** Commonness in the regional species pool was estimated on the scale of 0–3. The local community abundance was estimated between 0–8. The local seed bank is the number of individuals found at each site. The local seed rain is the standardized total yearly site seed rain.
(TXT)

Table S3 **The correlation between abundance in different species pools.** Results from Spearman's Rank correlation coefficient test, exploring the correlation between abundance in different species pools. VEG: Local community, RSP: Regional species pool, SR: Local seed rain and SB: Local seed bank.
(PDF)
