# Supplementary material for: Seed Rain and Seed Bank Reveal that Seed Limitation Strongly Influences Plant Community Assembly in Grasslands
Source: PLoS One. 2014 Jul 24;9(7):e103352. doi: 10.1371/journal.pone.0103352 (PMC4109992; doi:10.1371/journal.pone.0103352)
Supplement: Table S1 — The occurrence of species in different species pools. The occurrence of species in the regional species pool, the local propagule pool, the local community (vegetation), the seed rain and the seed bank. The number represent at how many of the 12 ex-arable fields in Nynäs, Sweden the species was found. (PDF) [file pone.0103352.s001.pdf]

Species	Regional species pool	Local propagule pool	Vegetation	Seed rain	Seed bank
Achillea millefolium	12	6	0	6	0
Achillea ptarmica	4	2	2	0	0
Actaea spicata	1	0	0	0	0
Agrostis capillaris/stolonifera	12	12	12	12	12
Agrimonia eupatoria	11	1	1	0	0
Ajuga pyramidalis	11	0	0	0	0
Alchemilla sp.	12	10	10	0	0
Alisma plantago-aquatica	2	0	0	0	0
Allium oleraceum	8	0	0	0	0
Allium vineale	1	0	0	0	0
Alopecurus geniculatus	2	0	0	0	0
Alopecurus pratensis	12	11	11	0	0
Anemone hepatica	5	0	0	0	0
Anemone nemorosa	11	2	2	0	0
Antennaria dioica	7	0	0	0	0
Anthoxanthum odoratum	12	8	7	6	2
Anthriscus sylvestris	12	8	7	2	0
Aquilegia vulgaris	1	0	0	0	0
Arabis hirsuta	2	1	0	0	1
Arctium minus	1	0	0	0	0
Arctium tomentosum	2	0	0	0	0
Arenaria serpyllifolia	5	0	0	0	0
Helictotrichon pratense	10	2	2	0	0
Helictotrichon pubescens	12	4	4	0	0
Artemisia vulgaris	5	0	0	0	0
Atriplex patula	2	0	0	0	0
Berberis vulgaris	7	0	0	0	0
Bidens tripartita	6	2	1	1	0
Bistorta vivipara	2	0	0	0	0
Briza media	10	5	5	0	0
Calamagrostis arundinacea	9	0	0	0	0
Calamagrostis canescens	7	0	0	0	0
Calluna vulgaris	10	5	0	5	1
Caltha palustris	1	0	0	0	0
Campanula persicifolia	12	0	0	0	0
Campanula rotundifolia	12	4	4	0	0
Campanula trachelium	1	0	0	0	0
Capsella bursa-pastoris	1	2	0	2	0
Carex acuta	2	0	0	0	0
Carex canescens	3	0	0	0	0
Carex caryophyllea	3	2	2	0	0
Carex cespitosa	2	0	0	0	0
Carex demissa	3	1	0	0	1
Carex digitata	6	1	1	0	0
Carex echinata	3	0	0	0	0
Carex hirta	9	9	7	1	6

**Species**	**Regional species pool**	**Local propa-gule pool**	**Vegetation**	**Seed rain**	**Seed bank**
Carex nigra	5	1	1	0	0
Carex ovalis	11	7	7	4	4
Carex pallescens	10	8	8	4	1
Carex panicea	9	6	4	1	3
Carex pilulifera	11	6	5	2	0
Carex spicata	12	8	6	1	2
Carex vesicaria	5	0	0	0	0
Carex vulpina	2	0	0	0	0
Cardamine amara	1	0	0	0	0
Carlina vulgaris	3	0	0	0	0
Carum carvi	7	6	6	2	0
Centaurea jacea	12	8	8	3	1
Cerastium fontanum	12	8	7	1	7
Chenopodium album	1	0	0	0	0
Chelidonium majus	2	0	0	0	0
Cirsium arvense	10	2	1	0	1
Cirsium palustre	10	3	3	1	0
Cirsium vulgare	7	2	1	1	0
Comarum palustre	3	0	0	0	0
Convolvulus arvensis	1	0	0	0	0
Convallaria majalis	7	0	0	0	0
Crepis praemorsa	1	0	0	0	0
Dactylorhiza incarnata	1	0	0	0	0
Dactylorhiza latifolia/sambucina	2	0	0	0	0
Dactylorhiza maculata	1	0	0	0	0
Dactylis glomerata	12	12	12	5	0
Danthonia decumbens	10	0	0	0	0
Deschampsia cespitosa	12	12	12	12	11
Deschampsia flexuosa	12	2	0	2	0
Dianthus deltoides	7	0	0	0	0
Eleocharis mamillata	1	0	0	0	0
Elytrigia repens	9	8	8	1	0
Epilobium adenocaulon	9	7	0	5	4
Epilobium montanum	3	0	0	0	0
Epilobium palustre	4	0	0	0	0
Erigeron acer	1	0	0	0	0
Eriophorum angustifolium	2	0	0	0	0
Erophila verna	1	0	0	0	0
Euphrasia stricta	5	3	3	0	0
Fallopia convolvulus	5	0	0	0	0
Festuca ovina	12	4	4	1	0
Festuca pratensis	12	12	12	12	0
Festuca rubra	12	12	12	5	0
Filipendula ulmaria	11	4	3	1	0
Filipendula vulgaris	11	6	6	0	0
Fragaria vesca	12	2	1	1	0

Species	Regional species pool	Local propagule pool	Vegetation	Seed rain	Seed bank
Fragaria viridis	2	0	0	0	0
Galium album	8	4	4	0	0
Galium aparine	9	2	2	0	0
Galium boreale	11	6	5	2	0
Galium palustre	8	2	1	1	0
Galeopsis speciosa	8	1	1	0	0
Galeopsis tetrahit	5	0	0	0	0
Galium uliginosum	7	5	5	0	0
Galium verum	12	10	10	0	0
Gentianella campestris	2	0	0	0	0
Geranium columbinum	2	0	0	0	0
Geranium molle	2	0	0	0	0
Geranium robertianum	4	0	0	0	0
Geranium sanguineum	2	0	0	0	0
Geranium sylvaticum	9	2	0	1	1
Geum rivale	12	9	8	4	0
Geum sp.	5	1	1	0	0
Glechoma hederacea	4	1	1	0	0
Glyceria fluitans	5	0	0	0	0
Gnaphalium sylvaticum	5	2	0	0	2
Helianthemum nummularium	12	0	0	0	0
Heracleum sp.	3	0	0	0	0
Hieracium sect. *Hieracium*	8	0	0	0	0
Hieracium sect. *Tridentata*	9	1	1	0	0
Hieracium umbellatum	10	0	0	0	0
Hypericum maculatum	12	4	4	0	1
Hypericum perforatum	11	2	2	0	0
Hypochaeris maculata	5	0	0	0	0
Iris pseudacorus	2	0	0	0	0
Juncus articulatus	7	8	0	2	8
Juncus bufonius	4	8	0	2	7
Juncus conglomeratus	8	0	0	0	0
Juncus effusus	12	11	2	8	11
Juncus filiformis	1	0	0	0	0
Juncus compressus/gerardii	2	1	0	1	0
Lamium purpureum	2	0	0	0	0
Laserpitium latifolium	3	0	0	0	0
Lathyrus linifolius	12	1	1	0	0
Lathyrus pratensis	12	12	12	1	1
Lathyrus vernus	1	0	0	0	0
Lemna minor	1	0	0	0	0
Leontodon autumnalis	12	12	12	9	2
Leucanthemum vulgare	12	3	2	1	0
Linum catharticum	6	0	0	0	0
Logfia arvensis	4	0	0	0	0
Lolium perenne	5	1	1	0	0

Species	Regional species pool	Local propagule pool	Vegetation	Seed rain	Seed bank
Lonicera caprifolium	1	0	0	0	0
Lonicera xylosteum	1	0	0	0	0
Lotus corniculatus	12	9	9	0	0
Luzula campestris	10	9	7	5	4
Luzula multiflora	10	6	6	3	0
Luzula pilosa	11	2	1	1	0
Lychnis flos-cuculi	1	0	0	0	0
Lychnis viscaria	7	0	0	0	0
Lysimachia vulgaris	7	0	0	0	0
Lythrum salicaria	4	1	0	1	0
Tripleurospermum perforatum	5	0	0	0	0
Matricaria suaveolens	7	0	0	0	0
Medicago lupulina	3	0	0	0	0
Melampyrum pratense	4	0	0	0	0
Melica nutans	7	0	0	0	0
Mentha arvensis	8	4	3	0	1
Moehringia trinervia	5	0	0	0	0
Monotropa hypopitys	1	0	0	0	0
Montia fontana	1	0	0	0	0
Myosotis laxa	9	0	0	0	0
Mycelis muralis	8	0	0	0	0
Myosotis arvensis	9	3	0	2	1
Myosoton aquaticum	1	0	0	0	0
Nardus stricta	2	0	0	0	0
Orchidaceae sp.	1	0	0	0	0
Origanum vulgare	1	0	0	0	0
Orthilia secunda	1	0	0	0	0
Oxalis acetosella	7	0	0	0	0
Paris quadrifolia	1	0	0	0	0
Peucedanum palustre	5	0	0	0	0
Persicaria hydropiper	10	0	0	0	0
Persicaria lapathifolia	3	1	0	1	0
Phalaris arundinacea	1	0	0	0	0
Phleum phleoides	2	0	0	0	0
Phleum pratense	12	12	12	7	5
Phragmites australis	4	0	0	0	0
Pilosella lactucella	8	1	1	0	0
Pilosella officinarum	12	3	3	1	0
Pimpinella saxifraga	12	9	9	2	0
Plantago lanceolata	11	8	8	5	1
Plantago major	10	8	6	5	4
Plantago media	4	0	0	0	0
Platanthera bifolia	6	0	0	0	0
Poa annua	6	6	1	3	2
Poa nemoralis	2	1	1	0	0
Poa trivialis	4	5	3	2	0

Species	Regional species pool	Local propa-gule pool	Vegetation	Seed rain	Seed bank
Poa pratensis/compressa	12	12	12	12	12
Polygala amarella	1	0	0	0	0
Polygonum aviculare	8	2	1	1	0
Polygonatum odoratum	4	0	0	0	0
Polygala vulgaris	11	1	1	0	0
Potentilla anserina	10	1	1	0	0
Potentilla argentea	11	1	0	0	1
Potentilla crantzii	7	1	1	0	0
Potentilla erecta	12	4	4	0	0
Potentilla reptans	12	9	8	7	2
Potentilla tabernaemontani	4	0	0	0	0
Primula veris	11	5	5	0	0
Prunella vulgaris	10	7	7	5	2
Pyrola chlorantha	1	0	0	0	0
Pyrola rotundifolia	1	0	0	0	0
Ranunculus acris	12	12	12	12	12
Ranunculus auricomus	10	12	10	10	4
Ranunculus bulbosus/repens	11	10	10	0	1
Ranunculus flammula	5	1	0	1	0
Ranunculus scleratus	1	0	0	0	0
Rhinanthus minor/serotinus	7	3	3	0	0
Ribes alpinum	8	0	0	0	0
Ribes uva-crispa	4	0	0	0	0
Rosa sp.	12	0	0	0	0
Rubus idaeus	12	0	0	0	0
Rubus saxatilis	6	1	0	0	1
Rumex acetosella	11	0	0	0	0
Rumex acetosa	12	12	12	9	3
Rumex crispus	10	4	4	0	1
Rumex longifolius	7	1	0	1	0
Sagina procumbens	3	7	1	4	5
Satureja acinos	2	0	0	0	0
Satureja vulgaris	2	0	0	0	0
Saxifraga granulata	5	1	0	1	0
Scirpus sylvaticus	6	0	0	0	0
Scleranthus annuus	8	0	0	0	0
Scorzonera humilis	5	1	0	1	0
Scrophularia nodosa	5	1	0	1	0
Scutellaria galericulata	1	0	0	0	0
Sedum acre	6	0	0	0	0
Sedum album	3	0	0	0	0
Hylotelephium telephium	12	1	0	1	0
Senecio sylvaticus	8	0	0	0	0
Senecio viscosus	7	0	0	0	0
Serratula tinctoria	2	0	0	0	0
Silene nutans	1	0	0	0	0

**Species**	**Regional species pool**	**Local propagule pool**	**Vegetation**	**Seed rain**	**Seed bank**
Solanum dulcamara	3	0	0	0	0
Solidago virgaurea	5	2	0	2	0
Sonchus arvensis	2	2	0	2	0
Spergula arvensis	3	0	0	0	0
Spergula morisonii	4	0	0	0	0
Stellaria graminea	12	12	12	8	12
Stellaria media	6	0	0	0	0
Succisa pratensis	9	2	2	0	0
Tanacetum vulgare	3	0	0	0	0
Taraxacum sect. Ruderalia	12	12	12	11	0
Thalictrum flavum	1	0	0	0	0
Thlaspi arvense	2	0	0	0	0
Thlaspi caerulescens	6	0	0	0	0
Thymus serpyllum	2	0	0	0	0
Tilia cordata	1	0	0	0	0
Tragopogon pratensis	2	0	0	0	0
Trifolium arvense	11	0	0	0	0
Trifolium aureum	7	0	0	0	0
Trientalis europaea	1	0	0	0	0
Trifolium hybridum	11	1	1	0	0
Trifolium medium	12	11	10	2	3
Trifolium pratense	12	12	12	6	3
Trifolium repens	12	12	12	11	10
Trifolium montanum	1	0	0	0	0
Triglochin palustre	1	0	0	0	0
Triticum aestivum	1	0	0	0	0
Tussilago farfara	7	3	0	3	0
Urtica dioica	11	2	0	0	2
Vaccinium myrtillus	11	2	0	1	1
Vaccinium vitis-idaea	10	1	0	1	0
Veronica agrestis/arvensis	2	3	0	2	2
Veronica beccabunga	3	0	0	0	0
Veronica chamaedrys	12	12	12	9	6
Veronica serpyllifolia/officinalis	12	12	8	9	12
Veronica scutellata	2	0	0	0	0
Verbascum thapsus	1	0	0	0	0
Vicia cracca	12	12	12	0	0
Vicia hirsuta	3	0	0	0	0
Vicia sepium	7	0	0	0	0
Vicia sylvatica	1	0	0	0	0
Vicia tetrasperma	1	0	0	0	0
Vincetoxicum hirundinaria	6	0	0	0	0
Viola arvensis	2	4	1	0	3
Viola canina	12	4	4	1	0
Viola hirta	2	0	0	0	0
Viola palustris	2	0	0	0	0

**Species**	**Regional species pool**	**Local propa-gule pool**	**Vegetation**	**Seed rain**	**Seed bank**
Viola riviniana	9	0	0	0	0
Viola tricolor	8	0	0	0	0
Senecio vulgaris	0	2	0	2	0
Myosurus minimus	0	2	0	0	2
Oenothera biennis	0	1	0	1	0
Digitalis purpurea	0	1	0	0	1
Conyza canadensis	0	2	0	1	1
Chenopodium polyspermum	0	7	0	2	6
Arabidopsis thaliana	0	2	0	1	1
Arabis glabra	0	1	0	0	1
Aethusa cynapium	0	1	0	1	0
Total number of species	**278**	**138**	**99**	**86**	**57**
