# Supplementary material for: Seed Rain and Seed Bank Reveal that Seed Limitation Strongly Influences Plant Community Assembly in Grasslands
Source: PLoS One. 2014 Jul 24;9(7):e103352. doi: 10.1371/journal.pone.0103352 (PMC4109992; doi:10.1371/journal.pone.0103352)
Supplement: Table S3 — The correlation between abundance in different species pools. Results from Spearman's Rank correlation coefficient test, exploring the correlation between abundance in different species pools. VEG: Local community, RSP: Regional species pool, SR: Local seed rain and SB: Local seed bank. (PDF) [file pone.0103352.s003.pdf]

Table S3. The correlation between abundance in different species pools. Results from Spearman's Rank correlation coefficient test, exploring the correlation between abundance in different species pools. VEG: Local community, RSP: Regional species pool, SR: Local seed rain and SB: Local seed bank.

	VEG-RSP		SR-RSP		SB-RSP		SR-VEG		SB-VEG	
	P	rho	P	rho	P	rho	P	rho	P	rho
Langmyra	**0.001**	0.690	**0.004**	0.571	0.963	0.016	**0.001**	0.772	0.571	0.192
Langpark	**0.004**	0.407	0.315	0.175	**0.011**	0.617	**0.006**	0.455	0.865	0.046
Langmaren	**0.001**	0.568	0.394	0.168	0.556	0.140	**0.027**	0.418	0.471	0.171
Lindviken	**0.001**	0.451	**0.001**	0.619	**0.008**	0.676	**0.006**	0.542	**0.041**	0.552
Lindvikenaker	**0.003**	0.414	0.233	0.305	0.687	0.118	**0.006**	0.635	0.298	0.299
LitselbyA	**0.001**	0.708	**0.004**	0.537	0.162	0.395	**0.001**	0.592	0.431	0.229
LitselbyB	0.191	0.173	0.494	0.140	**0.001**	0.787	**0.024**	0.441	**0.015**	0.563
Mellanstugan	**0.001**	0.771	**0.001**	0.730	0.189	0.335	**0.001**	0.803	0.133	0.380
Nyckelby	0.519	0.130	**0.003**	0.674	0.402	0.299	**0.006**	0.632	0.092	0.560
Nyckelbykulle	0.215	0.226	0.058	0.442	0.394	0.258	**0.029**	0.500	0.333	0.292
SandvikenA	**0.001**	0.505	**0.028**	0.422	**0.001**	0.525	**0.001**	0.615	0.698	0.093
SandvikenB	**0.011**	0.430	0.058	0.539	**0.001**	0.832	**0.005**	0.730	**0.010**	0.640
